# LIQUORICE: detection of epigenetic signatures in liquid biopsies based on whole-genome sequencing data

**DOI:** 10.1093/bioadv/vbac017

**Published:** 2022-03-23

**Authors:** Peter Peneder, Christoph Bock, Eleni M Tomazou

**Affiliations:** 1 St. Anna Children’s Cancer Research Institute (CCRI), Vienna 1090, Austria; 2 Doctoral School in Microbiology and Environmental Science, University of Vienna, Vienna 1030, Austria; 3 CeMM Research Center for Molecular Medicine of the Austrian Academy of Sciences, Vienna 1090, Austria; 4 Institute of Artificial Intelligence, Center for Medical Statistics, Informatics, and Intelligent Systems, Medical University of Vienna, Vienna 1090, Austria; 5 Ludwig Boltzmann Institute for Rare and Undiagnosed Diseases, Vienna 1090, Austria

## Abstract

**Summary:**

Fragmentation patterns of cell-free DNA reflect the chromatin structure of the cells from which these fragments are derived. Nucleosomes protect the DNA from fragmentation, resulting in decreased sequencing coverage in regions of open chromatin. LIQUORICE is a user-friendly software tool that takes aligned whole-genome sequencing data as input and calculates bias-corrected coverage signatures for predefined, application-specific sets of genomic regions. The tool thereby enables a blood-based analysis of cell death in the body, and it provides a minimally invasive assessment of tumor chromatin states and cell-of-origin. With user-defined sets of regions that exhibit tissue-specific or disease-specific open chromatin, LIQUORICE can be applied to a wide range of detection, classification and quantification tasks in the analysis of liquid biopsies.

**Availability and implementation:**

LIQUORICE is freely and openly available as a Python package and command-line tool for UNIX-based systems from bioconda. Documentation, examples and usage instructions are provided at http://liquorice.computational-epigenetics.org.

**Supplementary information:**

Supplementary data are available at *Bioinformatics Advances* online.

## 1 Introduction

The analysis of cell-free DNA (cfDNA) from blood and other bodily fluids—commonly referred to as liquid biopsy—holds great promise for cancer diagnostics and other applications (Cescon *et al.*, 2020; Heitzer *et al.*, 2019; Wan *et al.*, 2017). While most liquid biopsy research has focused on detecting somatic mutations and changes in copy number, recent studies demonstrated that fragmentation patterns of cfDNA can provide important complementary information based on fragment size distributions (Cristiano *et al.*, 2019; Mouliere *et al.*, 2018; Peneder *et al.*, 2021; van der Pol and Mouliere, 2019) and coverage signatures (Ivanov *et al.*, 2015; Peneder *et al.*, 2021; Snyder *et al.*, 2016; Sun *et al.*, 2019; Ulz *et al.*, 2016, 2019). Nucleosomes protect DNA from degradation, therefore cfDNA fragments can serve as ‘nucleosome footprints’ that reflect the epigenome of the cells from which the fragments are derived.

Building on these observations, we have recently developed, validated and applied a method for *liquid biopsy regions-of-interest coverage estimation* (‘LIQUORICE’). This method enables the analysis of chromatin accessibility in cfDNA samples based on whole-genome sequencing data. It calculates bias-corrected coverage signatures for sets of user-provided genomic regions—typically regions with tissue-specific or tumor-specific open chromatin that can be used to assess cell-of-origin and epigenetic cell states. We utilized this method to study patients with childhood cancers (Peneder *et al.*, 2021), which have low somatic mutation rates and are difficult to analyze with genetic methods. To make LIQUORICE readily available and useful for a broad range of researchers and applications, we now present the LIQUORICE software as user-friendly, efficient, robust and well-documented tool.

## 2 The LIQUORICE command-line tool and python package

LIQUORICE is available as an easy-to-use command-line tool that requires no programming skills. In addition, we provide an open-source Python package that allows users to adapt the LIQUORICE workflow for inferring epigenetic signatures from whole-genome sequencing data to their own needs and applications. Additional documentations, including installation instructions, notes on parameters and output files, usage examples and test data, are available from http://liquorice.computational-epigenetics.org. A detailed description of the LIQUORICE workflow is provided in the Supplementary Methods.

LIQUORICE takes four files as input: (i) an indexed BAM file containing aligned reads from paired-end whole-genome sequencing of a liquid biopsy sample; (ii) one or more BED files, each representing a set of regions of interest—such as DNase I hypersensitivity sites or enhancer regions specific for a tissue or tumor; (iii) the FASTA file of the reference genome; and (iv) a bigWig mappability file, which we provide for the hg19 and hg38 assemblies of the human genome and which is easily generated for other reference genomes. The selection of user-provided region sets depends on the application and typically includes regions of open chromatin of tissues or tumors that are relevant sources of cfDNA, which is obtained from blood plasma or other bodily fluids. Sets of tissue-specific and tumor-specific genomic regions can be obtained from public databases such as ENCODE (https://encodeproject.org) and the Regulatory Elements Database (Sheffield *et al.*, 2013). We provide an example of such data sources in Supplementary Figure S1.

**Fig. 1.**
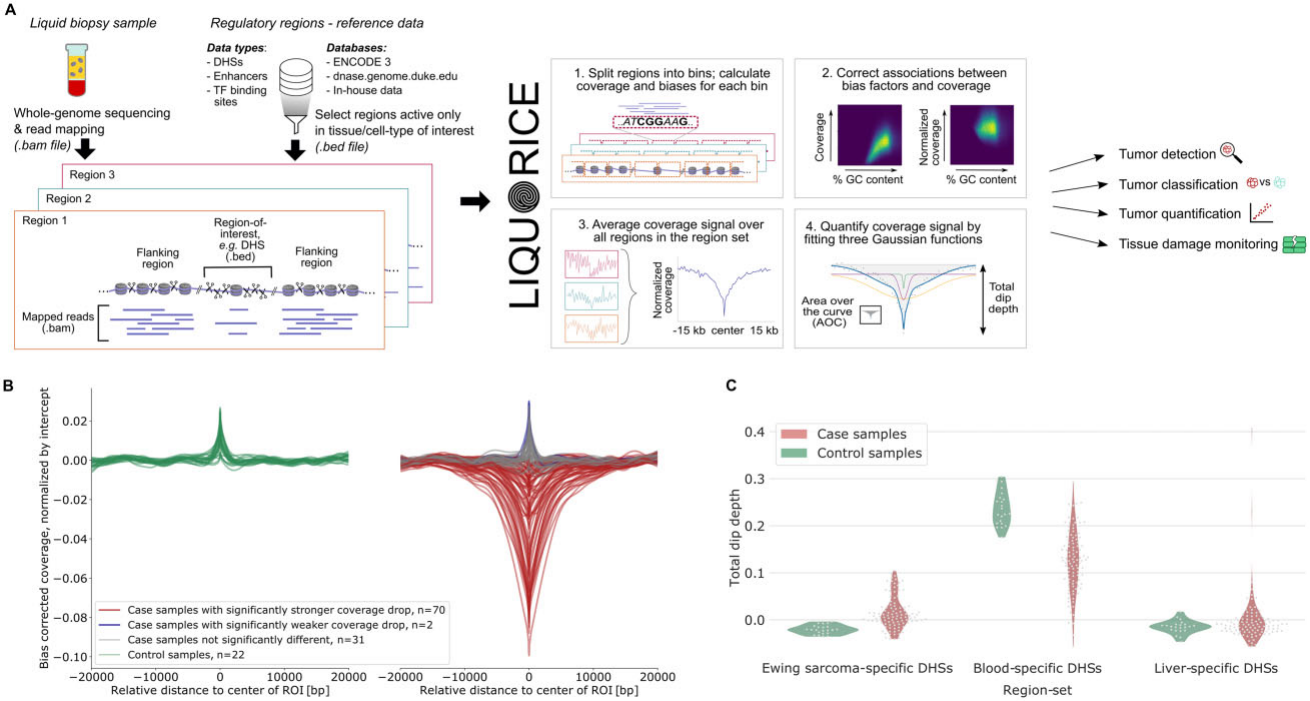
Overview of LIQUORICE’s workflow and output. (**A**) cfDNA fragments are ‘nucleosome footprints’, with fewer sequenced fragments (purple lines) in regions that are not well protected by nucleosomes (grey cylinders). LIQUORICE exploits this phenomenon by calculating bias-corrected coverage metrics from whole-genome sequencing data, focusing on a user-provided set of regions of interest. For better visualization, only three regions are shown here; in contrast, most LIQUORICE analyses use hundreds or thousands of regions of a given type (such as tissue-specific open chromatin regions or transcription factor binding sites). (**B**) Using LIQUORICE’s summary function, epigenetic signatures at regions-of-interest (here: Ewing sarcoma-specific DNase I hypersensitivity sites) can be compared between two groups of samples (here: patients with Ewing sarcoma and healthy controls). (**C**) The summary function generates violin plots of the signature strength across region sets and groups of samples. Patients with Ewing sarcoma have stronger tumor-specific signatures and weaker blood-specific signatures (the weakening of blood-specific signatures can be explained by the dilution of blood-derived cfDNA by the tumor-derived cfDNA). For several patients, liver damage caused by therapy toxicity is evident from the increased strength of a liver-specific signature. DHS, DNase I hypersensitivity site; TF, transcription factor; ROI, region of interest. Parts of this figure are adapted from Peneder, *et al.* (2021). Multimodal analysis of cell-free DNA whole-genome sequencing for pediatric cancers with low mutational burden. *Nat. Commun*., **12**, 1–16.

LIQUORICE provides an integrated solution for fragment analysis at genomic regions of interest in liquid biopsies based on whole-genome sequencing data of cfDNA. It corrects for potential biases (GC content, mappability and di/trinucleotide frequencies) and quantifies the coverage signal (Fig. 1A). Moreover, LIQUORICE creates tabular and visual summaries of the coverage signal across samples and genomic region sets. When samples from case and control groups are available, LIQUORICE can test individual samples for significant deviations from the control group (Supplementary Methods).

A typical LIQUORICE analysis runs in <20 min per sample on a personal laptop (Intel i7-8750H CPU, 16 GB RAM), or in about 3 min per sample on a single compute node with 25 cores. Individual samples are processed independently and are readily parallelized on a compute cluster. LIQUORICE analysis profits from sequencing depths in the range of 5× to 15× genomic coverage, especially when the region sets of interest are relatively small (e.g. less than a thousand regions with an average length of a kilobase). Nevertheless, we have successfully used LIQUORICE with sequencing depths as low as 1× and fewer than 500 regions of interest (Peneder *et al.*, 2021).

To our knowledge, LIQUORICE is the first user-friendly and broadly applicable tool for analyzing coverage signatures at user-defined regions based on whole-genome sequencing data from liquid biopsies.

## 3 Usage example

The following code shows how to install and run LIQUORICE, applying it to a cohort of patients with Ewing sarcoma as well as healthy controls (Peneder *et al.*, 2021). This example uses the human reference genome provided as a single FASTA file, Ewing sarcoma-specific DNase I hypersensitivity sites (Sheffield *et al.*, 2017) provided as BED files, and mapped sequencing reads for each sample provided as BAM files (https://ega-archive.org/studies/EGAS00001005127).

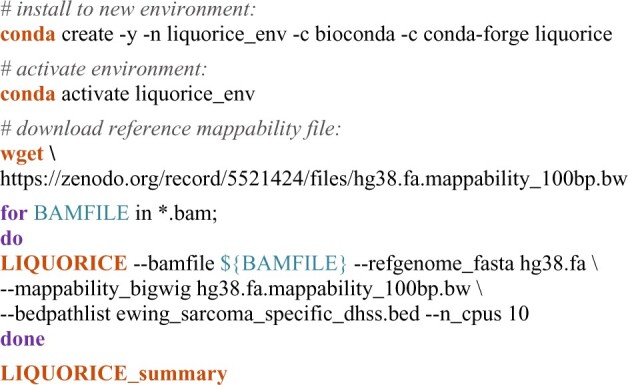

The resulting summary plots are shown in Figure 1B and C. The tabular summary (Supplementary Table S1) can be used as input to a machine learning classifier for tissue type or tumor detection and classification. We have recently demonstrated that such classifiers can accurately distinguish patients with Ewing sarcoma from healthy controls (ROC-AUC: 0.90), and from patients with other sarcomas (ROC-AUC: 0.92; Peneder *et al.*, 2021).

## 4 Conclusion

We present a user-friendly software tool for detecting tissue-specific and tumor-specific epigenetic signatures from liquid biopsies based on whole-genome sequencing data. LIQUORICE can be used for the detection, classification and quantification of signatures of any tissue or cell type, with application-specific, user-provided sets of genomic regions that represent characteristic epigenetic states of the relevant cell types (such as DNase I hypersensitivity sites or ChIP-seq peaks). We expect that LIQUORICE will be broadly useful for liquid biopsy analysis in oncology and beyond, for example for detecting and quantifying tumor DNA in cancers with few genetic alterations (based on tumor-specific regions of open chromatin); assessing on-target and off-target effects of cancer drugs (based on tumor-specific and organ-specific regions of open chromatin); and monitoring acute organ damage, for example in patients with sepsis and recipients of organ transplants (based on organ-specific regions of open chromatin).

## Supplementary Material

vbac017_Supplementary_Data
