# Supplementary material for: LIQUORICE: detection of epigenetic signatures in liquid biopsies based on whole-genome sequencing data
Source: Bioinform Adv. 2022 Mar 23;2(1):vbac017. doi: 10.1093/bioadv/vbac017 (PMC9710688; doi:10.1093/bioadv/vbac017)
Supplement: vbac017_Supplementary_Data [file vbac017_supplementary_data.zip › LIQUORICE application note - Supplementary Figure 1.pdf]

Supplementary Figures

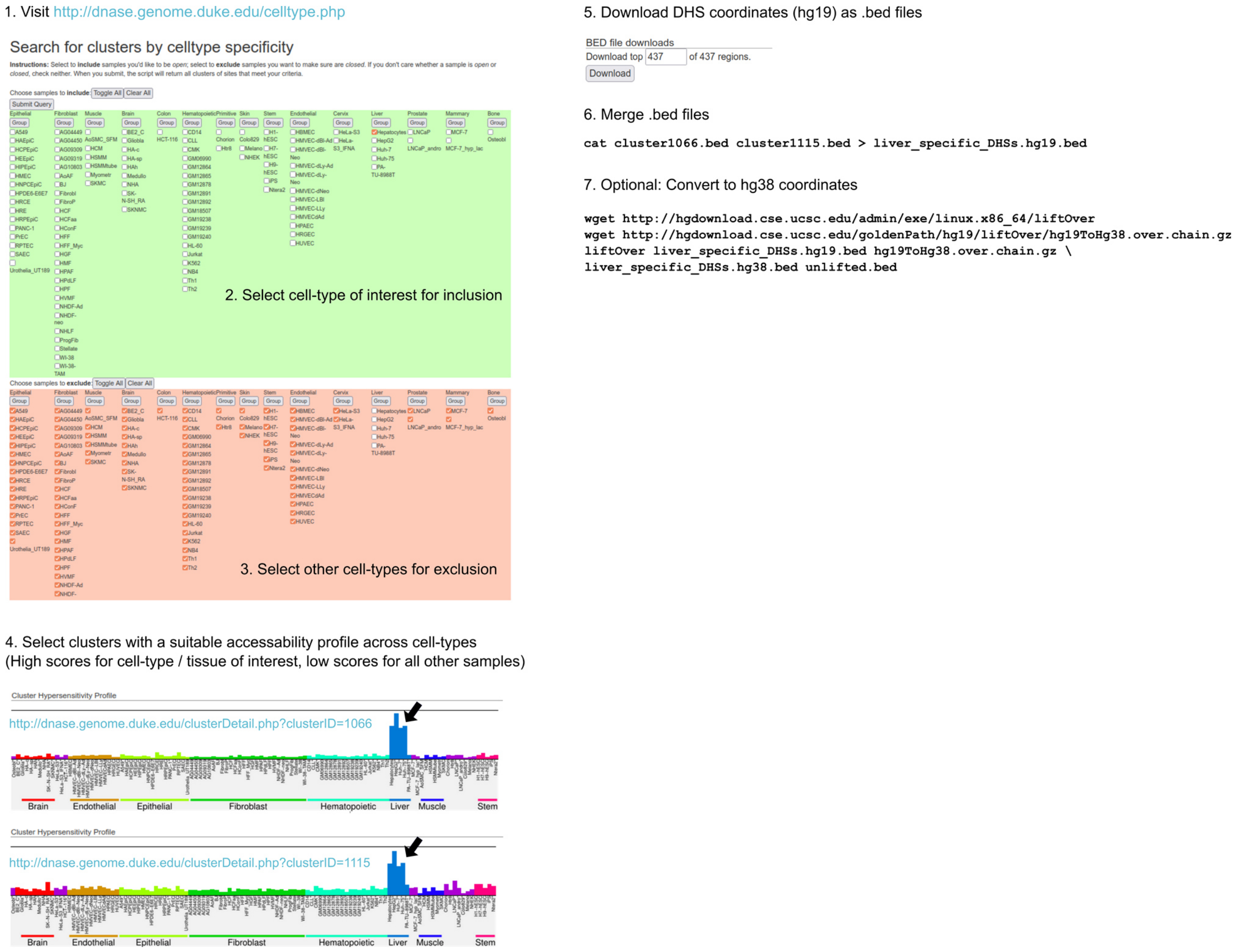

Supplementary Figure 1: Instructions on how to use the Regulatory Elements Database (http://dnase.genome.duke.edu) to obtain tissue-specific or cell-type specific DNase I hypersensitivity sites (DHSs) for analysis with LIQUORICE. The example shows how liver-specific DHSs can be obtained.
