# Supplementary material for: LIQUORICE: detection of epigenetic signatures in liquid biopsies based on whole-genome sequencing data
Source: Bioinform Adv. 2022 Mar 23;2(1):vbac017. doi: 10.1093/bioadv/vbac017 (PMC9710688; doi:10.1093/bioadv/vbac017)
Supplement: vbac017_Supplementary_Data [file vbac017_supplementary_data.zip › LIQUORICE application note - Supplementary Methods.pdf]

Supplementary Methods

LIQUORICE workflow

The following workflow is based on the previously published LIQUORICE method (Peneder et al., 2021) which has been adapted, extended and improved to make LIQUORICE an efficient, robust, and user-friendly tool.

LIQUORICE starts by analyzing the global fragment size distribution and the average genome-wide sequencing coverage for a given liquid biopsy sample, using representatively sampled fragments (the sampling is implemented using the deeptools library; Ramírez *et al.*, 2016). All further steps are performed separately for each user-provided region set.

To facilitate the analysis of regions with different lengths, each region in the region set is split into five bins, with sizes corresponding to 10%, 15%, 50%, 15%, and 10% of the region's total length. As the result, each region comprises five bins, irrespective of the region's length in basepairs (bp). Next, for each region, the adjacent genomic regions ("flanking regions"; default: 20 kb both upstream and downstream) are analyzed and split into bins (default: 500 bp). By default, each region comprises 85 bins.

Next, regions are filtered out and removed if they are not on autosomes, if they fall within a user-defined blacklist (default: Encode v2 blacklist, https://github.com/Boyle-Lab/Blacklist/blob/master/lists/hg38-blacklist.v2.bed.gz), or if they extend beyond the chromosome's borders.

For each bin, the genomic DNA sequence, the sequencing read mappability (based on mappability tracks calculated with the GEM software; Derrien *et al.*, 2012), and the sequencing coverage (normalized relative to genome-wide average) are determined. Optionally, the sequencing coverage can be corrected for copy-number aberrations.

LIQUORICE then employs a specialized approach to determine several bias factors for each bin, based on the mappability, GC-content, and di-/trincleotide frequency of the bin and its vicinity. This approach has been specifically designed for cancer detection in liquid biopsies. GC bias occurs at the fragment level, and usually some fragments only partially overlap with the bin. Therefore, also the GC content of a bin's flanking regions, not only the GC content of a bin itself may influence its coverage. The same applies to other biases, such as mappability bias. It has been shown that these flanking regions should not be ignored for an accurate bias correction (Benjamini and Speed, 2012), but the global fragment size distribution determines how strongly the properties of a bin's vicinity influence its coverage. Importantly, the global fragment size distribution of cell-free DNA differs between healthy individuals and patients with cancer (Mouliere *et al.*, 2018). LIQUORICE therefore specifically corrects for the effects of the global fragment size distribution on the coverage by determining biases at the fragment level. This procedure has been described in detail in Peneder *et al.*, 2021; a summary is provided here:

First, a coverage weight vector is calculated, which measures the degree to which biases at a given position relative to a bin's start are expected to influence the bin's coverage. Generally, biases that occur at positions within the bin or in close vicinity are expected to have a stronger influence than biases that occur further upstream or downstream. The fragment length has an impact on this phenomenon, because in samples with longer fragment sizes, biases further away from a bin can have stronger influence on its coverage than in samples with shorter fragment sizes (*Suppl. Methods Figure 1*).

The coverage weight vector is calculated using a random sample of fragment lengths from a sample's global fragment length distribution (default: n=1000). Then, iterating over every sampled fragment and

every possible starting position of the fragment relative to the bin, the coverage weight of every position that is covered by the fragment is increased by the fraction of the bin that is overlapped by the fragment (*Suppl. Methods Figure 1*, green dashed box). Since this calculation is independent of the bin's genomic context, the resulting vector is applicable to every bin with the appropriate length.

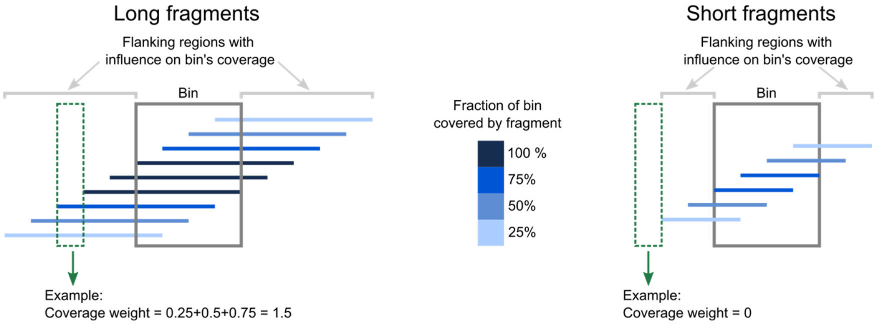

Suppl. Methods Figure 1: Illustration of how the coverage weight of a position is calculated in LIQUORICE. In samples with longer fragments (left) the flanking regions in which biases can have influence on the bin's coverage are wider and their influence is expected to be stronger than in samples with shorter fragments (right).

Second, the genomic context of every bin is considered to determine several bias vectors per bin (*Suppl. Methods Table 1*). The total bias factor of a given bin can then calculated be weighing the bias vector (strength of bias per position) by the coverage weight vector (degree to which bias at a position can impact coverage), summation of all elements in the resulting vector, and normalization to a range of 0-1 (by division with the sum of the coverage weight vector). For mappability-based bias factors, LIQUORICE uses a modified coverage weight vector in which all positions except the fragment start (forward mappability) or end (reverse mappability) are set to 0.

Suppl. Methods Table 1: Details on how each of the biases is calculated for a given position.

**Bias**	**Calculation (per position):**
GC-content	1 if "G" or "C", 0.461 if "N", else 0
Mappability (forward)	GEM mappability for a 5' to 3' read starting at this position
Mappability (reverse)	GEM mappability for a 3' to 5' read starting at this position
Mappability (max)	max(Mappability (forward), Mappability (reverse))
Dinucleotides (n=10)	1 if the base and the next base downstream correspond to the dinucleotide or its reverse complement, else 0
Trinucleotides (n=32)	1 if the base and the next two bases downstream correspond to the trinucleotide or its reverse complement, else 0

Third, after calculating the bias factors for every bin, LIQUORICE trains machine learning models to predict each bin's coverage, using its bias factors as features (*Suppl. Methods Table 2*). The dataset is split into two groups, and Histogram-based Gradient Boosting Regression Trees (scikit-learn; Pedregosa et al., 2011) are used to create out-of-fold predictions. By default, the five central bins that cover the core

region are excluded from the training set. For every bin (including the five central bins, and for all regions in the region set), LIQUORICE then corrects for the impact of the bias factors on the coverage by subtracting the predicted from the observed coverage values.

Suppl. Methods Table 2: Example of a training dataset for LIQUORICE's bias model

	Bin metadata (not used for training)					Label	Features									
							Mappability				Dinucleotides (n=10)			Trinucleotides (n=32)		
	Chromosome	*Start*	*End*	*Bin nr. (within region)*	*Bin size*	*Coverage*	*Forward mappability*	*Reverse mappability*	*max mappability*	*GC content*	*AA*	...	*CG*	*AAA*	...	*CGG*
n= n_{bins}*	chr1	7833460	7833960	0	500	0.198	0.999	0.996	0.999	0.332	0.259	...	0.005	0.120	...	0.002
	chr1	7833960	7834460	1	500	0.764	0.993	0.997	0.997	0.425	0.191	...	0.016	0.084	...	0.007
	...	...	...	...	...	...	...	...	...	...	...	...	...	...	...	...
	chr9	135177764	135178264	83	500	1.428	0.827	0.870	0.870	0.485	0.072	...	0.006	0.022	...	0.003
	chr9	135178264	135178764	84	500	1.130	0.996	1.000	1.000	0.560	0.101	...	0.022	0.025	...	0.017

$$^{*}n_{bins} = n_{regions} * \frac{< extend\ to >}{< bin\ size >}$$

Fourth, the bias-corrected coverage profiles are aggregated across all regions in the region set, and a model-based fitting approach is employed to quantify the changes in coverage around the center of the region. This approach is based on a combined model, consisting of an intercept and three Gaussian functions of different widths, each representing different aspects of nucleosome occupancy at gene-regulatory regions: (i) Transcription factor binding sites; (ii) enhancer or promoter regions; and (iii) large genomic elements such as super-enhancers. LIQUORICE then extracts several key metrics of the coverage signature from the fitted model: the amplitude and width of each of the three Gaussians, the Bayesian Information Criterion (representing goodness-of-fit), the intercept, as well as the total dip depth and total dip area. We have found that the latter two metrics are most useful for comparisons between samples.

Finally, LIQUORICE generates plots that show the corrected and uncorrected coverage profile and the fitted model.

LIQUORICE_summary

After LIQUORICE has been run on several samples (and, optionally, on multiple region sets), the LIQUORICE_summary command can be run as a convenient way to summarize the coverage signatures across samples and region sets (*Fig. 1B and C*). If both case and control samples are available, this command also assesses the differences between case and control samples based on prediction intervals.

"Case" samples are classified as "significantly different" from the control group if they have a score (dip area or dip depth) that is outside of the prediction interval of the control group. Here, the prediction interval is an estimate of an interval in which a future observation of a control sample will fall with a certain probability (default: 95%), given the control samples that have already been observed. The calculation assumes that the scores of the control samples follow a normal distribution. Therefore, LIQUORICE_summary performs the Shapiro-Wilk test for normal distribution and allows the user to assess the distribution of control scores via histograms and probability plots. Case samples that are classified as significantly different from the control group are shown in red or blue color in the overview plots. Additionally, LIQUORICE_summary produces a tabular overview in which the scores and information on significance for every sample are specified (*Supplementary Table 1*).

Open-source software used in LIQUORICE

We are grateful for the following open-source software used in LIQUORICE: Biopython (Cock *et al.*, 2009); deeptools and pyBigWig (Ramírez et al., 2016); joblib (https://joblib.readthedocs.io); scikit-learn (Pedregosa et al., 2011); matplotlib (Hunter, 2007); modin (Petersohn et al., 2020); numpy (Harris et al., 2020); pandas (McKinney, 2010); pybedtools and bedtools (Dale et al., 2011; Quinlan and Hall, 2010); pysam (https://github.com/pysam-developers/pysam); scipy (Virtanen et al., 2020); seaborn (Waskom, 2021); swifter (https://github.com/jmcarpenter2/swifter); and lmfit (Newville et al., 2014).

References *(Supplementary Methods):*

Benjamini,Y. and Speed,T.P. (2012) Summarizing and correcting the GC content bias in high-throughput sequencing. *Nucleic Acids Res.*, **40**, 1–14.

Cock,P.J.A. *et al.* (2009) Biopython: freely available Python tools for computational molecular biology and bioinformatics. *Bioinformatics*, **25**, 1422–1423.

Dale,R.K. *et al.* (2011) Pybedtools: a flexible Python library for manipulating genomic datasets and annotations. *Bioinformatics*, **27**, 3423–3424.

Derrien,T. *et al.* (2012) Fast Computation and Applications of Genome Mappability. *PLoS One*, **7**, e30377.

Harris,C.R. *et al.* (2020) Array programming with NumPy. *Nature*, **585**, 357–362.

Hunter,J.D. (2007) Matplotlib: A 2D graphics environment. *Comput. Sci. Eng.*, **9**, 90–95.

McKinney,W. (2010) Data Structures for Statistical Computing in Python. In, van der Walt,S. and Millman,J. (eds), *Proceedings of the 9th Python in Science Conference.*, pp. 56–61.

Mouliere,F. *et al.* (2018) Enhanced detection of circulating tumor DNA by fragment size analysis. **4921**, 1–14.

Newville,M. *et al.* (2014) LMFIT: Non-Linear Least-Square Minimization and Curve-Fitting for Python.

Pedregosa,F. *et al.* (2011) Scikit-learn: Machine learning in Python. *J. Mach. Learn. Res.*, **12**, 2825–2830.

Peneder,P. *et al.* (2021) Multimodal analysis of cell-free DNA whole-genome sequencing for pediatric cancers with low mutational burden. *Nat. Commun.*, **12**, 1–16.

Petersohn,D. *et al.* (2020) Towards Scalable Dataframe Systems. *Proc. VLDB Endow.*, **13**, 2033–2046.

Quinlan,A.R. and Hall,I.M. (2010) BEDTools: a flexible suite of utilities for comparing genomic features. *Bioinformatics*, **26**, 841–842.

Ramírez,F. *et al.* (2016) deepTools2: a next generation web server for deep-sequencing data analysis. *Nucleic Acids Res.*, **44**, W160–W165.

Virtanen,P. *et al.* (2020) SciPy 1.0: fundamental algorithms for scientific computing in Python. *Nat. Methods 2020 173*, **17**, 261–272.

Waskom,M.L. (2021) seaborn: statistical data visualization. *J. Open Source Softw.*, **6**, 3021.
